# Echocardiographic Calcium Score of Aortic Valve Correlates with Coronary Artery Calcium Score in Heterozygous Familial Hypercholesterolemia

**DOI:** 10.3390/life15030506

**Published:** 2025-03-20

**Authors:** Angelo Baldassare Cefalù, Emilio Nardi, Antonina Giammanco, Carola Maria Gagliardo, Carlo Maria Barbagallo, Ludovico La Grutta, Patrizia Toia, Federica Brucato, Chiara Scrimali, Teresa Maria Grazia Fasciana, Rossella Spina, Marina Lanza, Francesco Vitale, Davide Noto, Maurizio Averna

**Affiliations:** 1 Department of Health Promotion, Mother and Child Care, Internal Medicine and Medical Specialties “G. D’Alessandro” (PROMISE), University of Palermo, Via del Vespro 129, 90127 Palermo, Italy; abaldassare.cefalu@unipa.it (A.B.C.); emilio.nardi@unipa.it (E.N.); antonina.giammanco@unipa.it (A.G.); carola.gagliardo@libero.it (C.M.G.); carlo.barbagallo@unipa.it (C.M.B.); federicabrucato21@gmail.com (F.B.); chiara.scrimali@libero.it (C.S.); mariagrazia.fasciana@alice.it (T.M.G.F.); rossella.spina@libero.it (R.S.); mlanza127@gmail.com (M.L.); francesco.vitale@unipa.it (F.V.); 2 Section of Radiology, Department of Biomedicine, Neurosciences and Advanced Diagnostics (BiND), University Hospital “Paolo Giaccone”, 90127 Palermo, Italy; ludovico.lagrutta@unipa.it (L.L.G.); toiapatrizia@gmail.com (P.T.); 3 Institute of Biophysics (IBF), National Research Council (CNR), 90146 Palermo, Italy

**Keywords:** heterozygous familial hypercholesterolemia, CAC score, Agatston value, coronary calcium CT scan, echocardiogram, aortic valve calcification

## Abstract

Background: Patients with heterozygous familial hypercholesterolemia (HeFH) are at a high risk of atherosclerotic cardiovascular disease. The coronary artery calcification (CAC) score by the Ct-scan Agatston calcium score (ACS) > 100 classifies FH at a higher risk. The echocardiographic calcium score (ECS) evaluates aortic valve calcifications and is considered a good predictor of the atherosclerotic burden and cardiovascular outcome. Objective: To test the ECS as a predictor of ACS > 100 in a HeFH cohort. Methods: A coronary calcium CT scan with the calculation of ACS and an at rest-transthoracic echocardiogram with ECS evaluation were performed in 81 HeFH patients. Patients were divided into two groups according to the ACS: high-risk ACS patients (High-ACS) with Agatston value > 100 and low risk ACS patients (Low-ACS) with Agatston value ≤ 100. Patients were stratified according to ECS = 0 or ECS > 0. Results: High-ACS patients were older than Low-ACS patients; BMI, waist circumference, and blood systolic pressure were significantly higher (*p* < 0.001) in High-ACS patients. The ECS predicted an ACS > 100 with sensitivity = 0.84, specificity = 0.89, accuracy = 0.86, and precision = 0.76. Conclusions: The ECS could be a good surrogate of a coronary calcium CT scan for ACS evaluation in the specific subset of HeFH patients.

## 1. Introduction

Heterozygous familial hypercholesterolemia (HeFH) is a monogenic co-dominant hereditary disorder characterized by high plasma levels of low-density lipoprotein cholesterol (LDL-C). Individuals with HeFH face a tenfold increased risk of atherosclerotic cardiovascular disease (ASCVD) compared to unaffected subjects [1]. Worldwide, the prevalence of HeFH has been estimated as being as high as 1:313 [2].

The relationship between HeFH and atherosclerotic coronary disease is well established, while the association between HeFH and aortic valve calcification is a topic of ongoing investigation because of the association of this condition with an increase in all-cause and cardiovascular mortality [3].

Cardiovascular (CV) risk stratification in HeFH is crucial for establishing appropriate treatment and management strategies. Different diagnostic imaging tests have been proposed in recent years to identify preclinical atherosclerosis in HeFH patients [3]. Measurement of coronary artery calcification (CAC), by computed tomography (CT), is the most used technique to detect preclinical coronary atherosclerosis [4,5,6,7], providing a good estimate of the atherosclerosis burden. A coronary calcium CT scan allows the measure of the Agatston calcium score (ACS). The ACS (0, 1–100, 101–400, >400) classifies patients in four CV risk categories (1%, <10%, 10–20%, and >20%) of probability of suffering a major adverse cardiovascular event (MACE) in the next 10 years [8]. However, the extensive use of it is hindered by costs, the availability of CT scanners, and radiation exposure [9]. In patients with HeFH, ACS > 100 has been proven to be a good predictor of MACE [10]. In addition to CAC scoring by ACS, carotid ultrasound (US) and coronary CT have been proposed for CV risk stratification and as predictors of MACE in HeFH patients [11,12].

Echocardiography is widely used for detecting a calcified lesion of the valvular apparatus and ascending aorta [13] but it is limited in the quantitative estimate of calcifications. However, echocardiographic evaluation of calcifications of the mitral annulus, papillary muscles, aortic valve, and ascending aorta is able to predict: (i) the burden of coronary and cardiac calcifications, (ii) the presence of coronary plaques, (iii) the cardiovascular outcomes [12,13]. Nevertheless, current guidelines do not incorporate echocardiography as a diagnostic tool for CV risk prediction in HeFH patients [14]. The present study was designed to evaluate the reliability and performance of echocardiography both as a predictor of the CAC score measured by a coronary calcium CT scan, and to stratify the subclinical CV risk in HeFH-patients.

## 2. Materials and Methods

This observational study was conducted in the Lipid Outpatients Clinic at the University Hospital “Paolo Giaccone” in Palermo. Previously characterized HeFH patients were included in the study [15].

A total number of 81 HeFH patients with untreated LDL-C levels > 4.88 mmol/L at diagnosis were enrolled. Patients diagnosed with aortic stenosis, bicuspid aortic valve, uncontrolled hypertension, and previous MACE were excluded from the study. Homozygous FH (HoFH) patients were also excluded.

The study was approved on the 9 December 2025 by the Institutional Ethics Committee (protocol number 11/2015). All patients provided informed consent to undergo a coronary calcium CT scan and a transthoracic echocardiogram within 2 months.

### 2.1. ACS Measurement by Coronary Calcium CT Scan

All enrolled patients were scheduled to undergo a coronary calcium CT scan for a CAC score calculation, using a standard protocol with a 128-slices- scanner with administration of a radiation dose of <1.5 mSv. Acquired data were subsequently transferred to a dedicated workstation with dedicated post-processing software. The CAC was quantified over the entire epicardial coronary system using the Agatston method (Agatston calcium score—ACS) as previously described [16] by a single radiologist with extensive experience in heart CT. The entire enrolled cohort of HeFH patients was divided into two groups according to the CAC score: (a) ACS high risk (High-ACS) patients with an Agatston value > 100 and (b) ACS low risk (Low-ACS) patients with an Agatston value ≤ 100. The cut-off of 100 was chosen according to previous studies identifying this value as a good predictor of a severe FH phenotype (high relative and absolute risk of coronary heart disease events and mortality) [8,10].

### 2.2. Echocardiographic Calcium Score (ECS)

Within 2 months of the CT evaluation, a standard transthoracic echocardiographic examination was planned for all patients, using an Aplio i700 ultrasound machine—Canon Medical System Europe, Amstelveen, The Netherlands. Echocardiographic examination was focused on the study of semilunar aortic valve; 2D-images were acquired, with the integration of a Doppler study. Maximum velocity in aorta, and maximum and mean gradient were also recorded. For the evaluation of AVC, a previously described echocardiographic calcium score (ECS) was used [17,18]. Briefly, the scoring system considers four classes: 0 = Absent (no calcifications), 1 = Mildly calcified (isolated small spots, cusps thickness > 2 mm and/or increased reflectivity), 2 = Moderately calcified (multiple bigger spots, cusps thickness >4 mm and/or diffuse), 3 = Heavily calcified (extensive thickening/calcification of all cusps/marked reflectivity). After image acquisition, all images were stored in DICOM format on a hard disk. A random sample of 10 recorded exams was selected from each group to evaluate the variability within and between observers. Two experienced ultrasound physicians conducted independently two-dimensional evaluations of aortic valve calcifications.

Following a 2-week interval, the same physician reanalyzed the recorded echocardiograms of the selected subjects. Intra-observer variability was assessed by comparing the measurements from the first physician’s two assessments, while inter-observer variability was evaluated based on the results from both physicians. The intra-observer and inter-observer correlation coefficients were considered excellent. The intra-observer and inter-observer variabilities were higher than 0.8.

**Figure 1:**
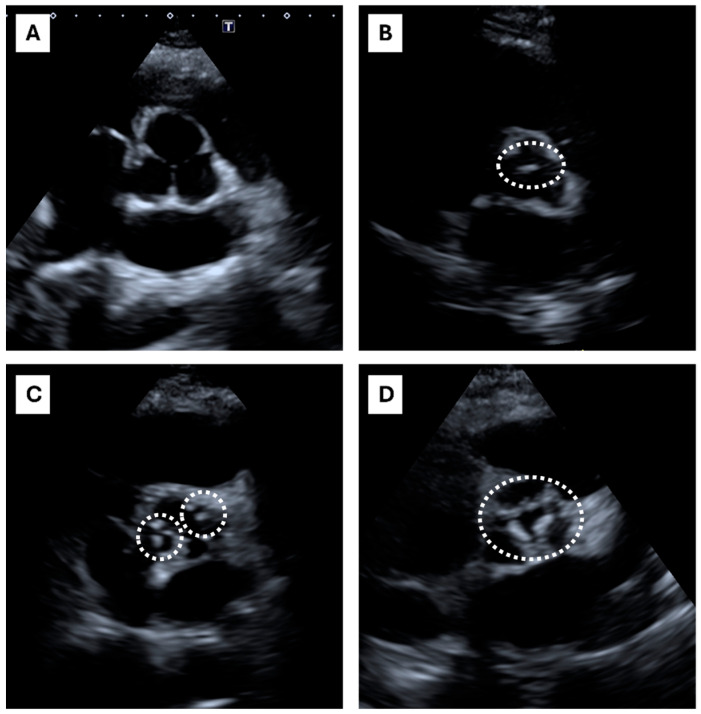
Echocardiographic calcium score: echocardiographic short-axis parasternal view. The figure shows the echocardiographic short-axis parasternal view, focused on the aortic valve. At the center of each box, aortic valve and cusps are shown. The dashed lines encircle aortic valve calcifications. The ECS scoring system considers four classes: (**A**) ECS = 0, Absent (no calcifications); (**B**) ECS = 1, Mildly calcified (the dashed line encircles an isolated small spot), (**A**,**C**) ECS = 2, Moderately calcified (multiple bigger spots are encircled by dashed lines), (**D**) ECS = 3, Heavily calcified (extensive thickening/calcification of all cusps).

Typical ECS patterns are presented in Figure 1.

### 2.3. Statistics

Patients were divided into two or more groups according to their ACS and ECS. Due to the differences in age among groups, all calculations were age- and gender-adjusted: numerical variable differences were evaluated by ANOVA analysis using age and gender as covariates; for dichotomic variables, a logistic regression analysis was performed. ACS high-risk prediction was assessed by multiple logistic regression analysis (MLRA) using age, male gender, BMI, systolic blood pressure, systolic blood pressure, minimal ABI, and ECS as predictors. When one-to-one group comparison was required, MLRA was run using only the two groups under investigation. If more than two groups were involved, as in the ECS x ACS cross-tabulations, one-by-one comparisons were calculated by ANOVA post hoc Bonferroni tests. The predictive power of the ECS to predict the ACS was also evaluated by ROC curva analysis with a pROC package. All the calculations were performed by R scientific software running within an Rstudio suite graphical interface, R version 4.3.2 (The R Foundation for Statistical Computing, Institute for Statistics and Mathematics, Vienna University of Economics and Business, Vienna, Austria. https://www.r-project.org/).

## 3. Results

**Figure 2:**
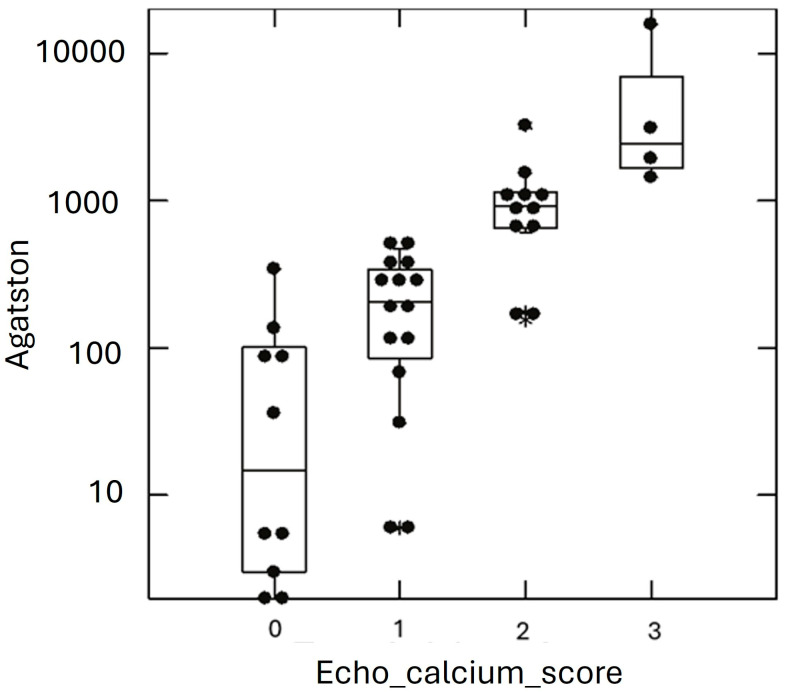
ACS values in HeFH cohort according to ECS classes. Graphical representation of individual subjects (black dots) Agatston calcium score (ACS) values (y axis) according to the echocardiographic calcium score (ECS) classes (x axis). Star symbol represents outliers.

A total of 45 out of 81 patients presented an ECS = 0, 21 out of 81 presented an ECS = 1, 11 out of 81 presented an ECS = 2, and 4 out of 81 presented an ECS = 3. Typical ECS patterns are presented in Figure 1. Clinical characteristics of HeFH subjects are shown in Table 1. The table shows the study cohort divided according to the ACS. High-ACS patients (ACS > 100) were older than the Low-ACS (ACS ≤ 100) (*p* < 0.001), and had FH diagnosed later in life (*p* < 0.001); lipid-lowering therapy (LLT) was also started later (*p* < 0.001). The High-ACS patients were also more obese (both BMI and waist circumference were significantly higher) and had higher blood systolic pressure (*p* < 0.001). A strong linear correlation was found between the ACS and ECS (Figure 2).

Next, we evaluated the performance of the ECS as a predictor of the CT scan-CAC score, expressed as ACS > 100. We found a clear concordance between the two measures; in detail, the ECS predicted an ACS > 100 (sensitivity = 0.84, specificity = 0.89, accuracy = 0.86, precision = 0.76) (see Table 2).

Table 3 shows the differences between the patients with concordant (Low ACS/Low ECS risk and High ACS/High ECS risk), and discordant (Low ACS/High ECS and High ACS/Low ECS) risk prediction. The discordant groups showed somehow intermediate features in comparison with the concordant Low ACS/Low ECS risk and High ACS/High ECS risk groups. In detail, age, waist circumference, and blood pressure were significantly higher in the discordant group in comparison with the Low ACS/Low ECS risk group.

Age per each decade of life and ECS > 0 were found to be an independent risk factor for a high ACS value (see Table 4).

## 4. Discussion and Conclusions

Patients either clinically or genetically diagnosed with HeFH are considered at high cardiovascular risk due to the increased incidence of premature atherosclerotic cardiovascular disease (ASCVD) [19,20].

While various scores are commonly used to stratify the ASCVD risk in non-FH patients, such as the Framingham Risk Score, the Pooled Cohort Equation, and the European Systematic Coronary Risk Evaluation (SCORE) [21,22,23], these tools have not been specifically developed for FH patients. Consequently, their applicability might be inaccurate in this peculiar setting. Position statements on HeFH highlight the necessity of assessing cardiovascular risk to guide therapy and with this aim specific scores for FH, such as the Montreal-FH-SCORE (MFHS), had been validated [24,25]. This score considers only clinical and biochemical values. According to the latest guidelines, the comprehensive management of the FH patient must integrate this score with imaging assessment [26]. Imaging allows us to identify an established atherosclerotic disease and allows the follow-up for several years. It also may detect subclinical atherosclerosis thanks to the development of new techniques. Bilateral carotid US, echocardiography, and the coronary calcium CT scan are all established imaging techniques to evaluate the atherosclerosis burden [24,26].

While this approach is suggested in heterozygous FH patients, it is strongly recommended in homozygous patients due to their even higher intrinsic cardiovascular risk [24].

A coronary calcium CT scan for an CAC score calculation is mentioned in the latest European Atherosclerosis Society Consensus Statement on HoFH patients as an established diagnostic method for the prediction of subclinical ASCVD [14]. According to the International Atherosclerosis Society (IAS), a CAC score value > 100 by the Agatston method is also used to define a severe phenotype of FH patients [10]. The primary advantage of ACS lies in quantifying coronary calcium deposits. Conversely, its elevated cost and the radiologic risk exposure limit its widespread use for all patients [8].

Aortic valve calcification is highly prevalent in elderly patients, and it is strongly related to atherosclerosis, sharing a common CV risk factor profile with atherosclerosis, characterized by hypertension, diabetes, cigarette smoking, and hypercholesterolemia [27]. The mechanism underlying the formation of cardiac calcifications is not yet fully understood; however, it is known that it is not only a passive phenomenon caused by tissue degeneration, but it also involves an active and intricate biological interplay of different cellular components [28]. Moderate or severe AVC has been shown to be an independent predictor of mortality, hence the importance of early detection and accurate quantification of valve calcifications [29].

The ECS score is regarded as a reliable method for qualitative estimation of an AVC. The disadvantage of the ECS lies in the lack of a quantitative estimate of calcifications [18].

Moreover, the echocardiogram overall lacks accuracy to discriminate calcification from fibrosis, particularly when a single lesion occurs. However, multiple calcifications involving all cusps may be easily distinguished from fibrosis.

The present study tested the ECS as an alternative to an ACS in assessing the CV risk of HeFH patients. Few studies proposing a correlation between the ECS and ACS have been published to our knowledge [18,30]. This study identified a strong correlation between the ECS and non-coronary calcium score but a weak correlation between the ECS and CAC. These studies were not conducted on HeFH patients, and a different ACS cutoff (ACS > 400) was chosen.

In our study, age per each decade of life and an ECS > 0 were found to be independent risk factors for a high ACS value. It evidenced a good sensitivity and specificity of ECS > 0 in predicting an ACS > 100. In a small number of patients, the CV risk was attributed discordantly according to the ACS or ECS (eight patients in the Low ACS/High ECS group and three patients in the High ACS/Low ECS group). These patients presented intermediate clinical features in comparison with concordant categories (Low ACS/Low ECS risk and High ACS/High ECS risk). The data suggest that the discordant categories are somehow at intermediate risk between the concordant low (Low/Low risk) and high (High/High risk) categories and that the ECS can help to individuate some patients who would otherwise be considered to be at lower risk by the CAC score. This topic should be investigated on a larger sample of patients. Nevertheless, the main limitation of our study consists of a lack of prospective data for the evaluation of the CV risk over time.

In conclusion, our preliminary results suggest that a “calcium-targeted” echocardiographic examination could be a good alternative to an ACS for ASCVD risk prediction in HeFH, providing in a short time comparable results to the ACS, reducing radiation exposure and costs for the public health system. However, further studies are necessary to confirm our observations. An ECS assessment could be particularly useful, especially in the follow-up of young HeFH patients (<50 years old), who rarely present valve calcifications, usually associated with aging and sclerosis. If supported by further evidence, ECS use could be proposed also for cardiovascular risk stratification in non-FH patients at a moderate/high CV risk.

## Figures and Tables

**Table 1:** Characteristics of the HeFH cohort according to the CAC score expressed as ACS.

Parameters	ACS ≤ 100	ACS > 100	*p*-Value ^1^
Cardiovascular risk based on CAC score	Low	High	-
Number of subjects	52	29	-
Age (yrs)	45.77 ± 12.29	62.69 ± 8.55	<0.001
Gender (M/F)	(24/28)	(11/18)	0.475
Smoking (No/Former/Active)	(9/3/17)	(14/10/28)	0.576
Age of FH diagnosis (yrs)	22.08 ± 13.62	33.57 ± 14.06	<0.001
Family history of CVD (Y/N)	(25/27)	(14/15)	0.927
Hypertension (Y/N)	(6/46)	(13/16)	0.297
Diabetes (Y/N)	(2/50)	(3/26)	0.729
Age at start of LLT (yrs)	26.21 ± 12.63	37.52 ± 14.66	<0.001
Clinical examination			
BMI (kg/m^2^)	26.36 ± 5.27	29.16 ± 4.73	0.007
Waist circumference (cm)	92.84 ± 15.9	99.24 ± 11.1	0.024
S.B.P. (mm/Hg)	121.81 ± 14.26	137.86 ± 23.97	<0.001
D.B.P. (mm/Hg)	73.38 ± 10.47	77.21 ± 13.11	0.083
Tendon xanthoma (Y/N)	(4/48)	(8/21)	0.260
Corneal arcus (Y/N)	(4/48)	(15/14)	0.128
Biochemical parameters			
Total cholesterol (mmol/L)	6.06 ± 1.83	5.69 ± 1.77	0.292
Triglyceride (mmol/L)	1.20 ± 0.57	1.22 ± 0.58	0.821
HDL cholesterol (mmol/L)	1.41 ± 0.36	1.49 ± 0.35	0.267
LDL cholesterol (mmol/L)	4.11 ± 1.65	3.65 ± 1.62	0.153
Lipid-lowering therapy			
Statins (Y/N)	(41/10)	(27/2)	0.407
Statins + Ezetimibe (Y/N)	(41/10)	(27/2)	0.407
PCSK9i Mab (Y/N)	(4/48)	(9/20)	0.493
Instrumental measures			
Coronary calcium CT scan			
ACS (range)	0 (0–80)	384 (101–15 k)	<0.001 ^2^
Echocardiography			
ECS > 0 (Y/N)	(8/44)	(26/3)	<0.001
V_max ^3^	1.38 ± 0.05	1.61 ± 0.07	0.022

ACS: Agatston calcium score, ECS: echocardiographic calcium score. ^1^ age and gender adjusted ANOVA for numeric variables, age and gender adjusted logistic regression for dichotomic variables. ^2^ after log transformation of Agatston value. ^3^ maximum aortic velocity registered with continuous Doppler. Y/N: yes/not. CVD: cardiovascular disease. BMI: body mass index. S.B.P.: systolic blood pressure. D.B.P.: diastolic blood pressure.

**Table 2:** Performance indices of ECS in predicting ACS > 100 at coronary calcium CT scan.

Specificity	0.846
Sensitivity	0.897
Accuracy	0.864
Precision	0.765
False positive rate	0.154
True positive rate	0.897
False negative rate	0.103
True negative rate	0.846

Estimation of echocardiographic score as predictor of the high risk (ACS > 100 at coronary calcium CT scan) subjects. ACS: Agatston calcium score, ECS: echocardiographic calcium score.

**Table 3:** HeFH cohort according to ECS and ACS values.

Parameters	ECS = 0 and ACS ≤ 100	ECS = 0 and ACS > 100	ECS > 0 and ACS ≤ 100	ECS > 0 and ACS > 100	*p*-Value ^1^
Risk according to ACS	Low	High	Low	High	-
Risk according to ECS	Low	Low	High	High	-
N of subjects	44	8	3	26	-
Age (years)	43.52 ± 11.80	58.12 ± 6.40	58.33 ± 3.05	63.12 ± 8.86 *	0.005
Male gender	0.45	0.50	1.00	0.30	0.995
BMI (kg/m^2^)	26.16 ± 5.09	28.87 ± 2.02	27.61 ± 6.6	29.19 ± 4.98 **	0.005
WC (cm)	102.73 ± 10.68	106.33 ± 7.37	101.57 ± 9.61	107.19 ± 12.35 *	0.033
S.B.P. (mm/Hg)	119.86 ± 13.62	135.33 ± 17.47 *	132.5 ± 13.68	138.15 ± 24.87 **	<0.001
D.B.P. (mm/Hg)	72.25 ± 10.16	88 ± 24.25 *	79.63 ± 10.54 *	75.96 ± 11.4	0.071
Hypertension	0.068	0.333 *	0.375	0.462 *	0.002
Diabetes mellitus	0.045	0	0	0.115	0.529
Tendon xanthoma	0.045	0	0.25	0.308 *	0.018
Corneal arcus	0.091	0	0	0.577	<0.001
CVD Family history	0.545	0.333	0.125	0.5	0.165
Age of FH diagnosis (yrs)	19.59 ± 12.72	23 ± 3.46 *	35.75 ± 10.25	34.84 ± 14.35 *	<0.001
TC (mmol/L)	236.68 ± 74.47	249.67 ± 84.71	231 ± 52.21	218.12 ± 67.95	0.264
TG (mmol/L)	102.64 ± 46.53	104.67 ± 33.53	119.25 ± 66.78	107.68 ± 52.99	0.768
HDL- C (mmol/L)	55.2 ± 14.24	59.33 ± 8.02	53.63 ± 13.33	57.76 ± 14.13	0.271
LDL- C (mmol/L)	160.95 ± 66.61	169.4 ± 73.88	153.53 ± 51.42	138.82 ± 62.46	0.132

Analysis of patients grouped according to Agatston calcium score (ACS) > 100 and echocardiographic calcium score (ECS) > 0. Columns 2 and 5 represent concordant groups (both scores identified low-risk and high-risk patients). Columns 3 and 4 represent discordant groups (scores are divergent in assigning the risk). ^1^ age and gender adjusted ANOVA for numeric variables, age and gender adjusted logistic regression *p* value for dichotomic variables.ECS = echocardiogram score. WC: waist circumference (cm). BMI: body mass index. S.B.P.: systolic blood pressure. D.B.P.: diastolic blood pressure. CVD: cardiovascular disease. TC: Total cholesterol. TG: triglycerides. HDL- C: HDL-cholesterol. LDL- C: LDL-cholesterol. One-to-one comparisons by Bonferroni post hoc tests for ANOVA, by one-to-one analysis for MLRA (see methods); * = *p* < 0.05, ** = *p* < 0.005 vs. Low/Low-risk group.

**Table 4:** Independent predictors of high ACS CV risk by coronary calcium CT scan.

Parameters	Odd Ratio (95% CI Interval)	*p*-Value ^1^
Age (×10 years) ^2^	14.48 (3.78–86.8)	0.003
Echocardiographic calcium score > 0	0.8 (1–4.42)	<0.001

Variables not entering the model: male gender, BMI, systolic blood pressure, minimal ABI. ^1^ multiple regression *p*-value. ^2^ The row shows an increasing of the odds ratio for every 10 years of age.

## Data Availability

Dataset available on request from the authors.

## Abbreviations

HeFH	Heterozygous familial hypercholesterolemia
HoFH	Homozygous familial hypercholesterolemia
ASCVD	Atherosclerotic cardiovascular disease
AVC	Aortic valve calcifications
CV	Cardiovascular
CAC score	Coronary artery calcium score
CT	Computed tomography
MACE	Major adverse cardiovascular event
ACS	Agatston calcium score
ECS	Echocardiographic calcium score
